# Parental Monitoring of Early Adolescent Social Technology Use in the US: A Mixed-Method Study

**DOI:** 10.1007/s10826-023-02734-6

**Published:** 2023-12-18

**Authors:** J. Maya Hernandez, Elana Pearl Ben-Joseph, Stephanie Reich, Linda Charmaraman

**Affiliations:** 1 School of Social Ecology, University of California, Irvine, Irvine, CA USA; 2 School of Public Health, Brown University, Providence, RI USA; 3 School of Education, University of California, Irvine, Irvine, CA USA; 4 Wellesley Centers for Women, Wellesley College, Wellesley, MA USA

**Keywords:** Early adolescence, Problematic technology use, Parental digital media monitoring, Family closeness, Mixed methods

## Abstract

Parental monitoring of digital media use evolves throughout childhood and adolescence and become increasingly important when autonomy seeking on social technologies emerges during early adolescence. We investigate a survey cohort of 248 US parents of early adolescents and their parental media monitoring behaviors (i.e., restrictive, active, deference), the family context (i.e., closeness and parent technoference), and perceptions of child’s problematic internet use. Using an explanatory mixed methods approach, the results of this study reveal that restrictive parental monitoring of adolescents’ digital media use is positively associated with child’s problematic internet use. However, active and deference monitoring are not associated with early adolescent problematic internet use and positively associated with family contexts. Familial closeness and technoference are consistent negative and positive correlates, respectively, of perceived problematic use among early adolescents. Qualitative interviews with a subset of 31 parents reveal while most parents report restrictive behaviors, multiple techniques (e.g., active, surveillant, and deference) are also leveraged when navigating their child’s online behaviors. Parents tended to converge on the same types and reasons for restrictive monitoring of media, whereas for other approaches the reasons behind their decision-making were quite divergent. The implication of this study is that parental media monitoring behaviors during early adolescence are rapidly evolving and not confined to a singular strategy, often related to prevention of or intervention on their child’s online behavior patterns. Understanding the family dynamics and parent involvement of adolescent’s digital media use remains critical in prevention of child’s problematic behaviors and promotion of positive online behaviors.

## Highlights

- Restrictive media monitoring is positively associated with parental perceptions of early adolescent’s problematic internet use.
- Active and deference monitoring is associated with family characteristics, but not early adolescent’s problematic online behaviors.
- Parental media monitoring strategies are constantly shifting depending on the child, context, and insecurities about effectiveness of their approaches.
- Family closeness and parental device use are also important indicators of early adolescent online behaviors.

Widespread use and relative affordability of mobile devices, such as smartphones, have allowed for the adoption of social technology (e.g., social media platforms, internet forums, interactive gaming) at increasingly younger ages. Recent reports show that children are engaging with social networking sites as early as 10 years old or younger (Charmaraman et al., 2022b; Rideout et al., 2022). The increasingly ubiquitous use (Rideout et al., 2022), and the difficulties in regulating this use, are concerning to parents. In response, researchers are exploring the effects of social technology in the context of family context and behaviors (O’Keeffe & Clarke-Pearson, 2011). Parents who are attempting to keep pace with the proliferation of social technology are faced with dual challenges of tracking their children’s online time as well as preventing negative online behaviors (and promoting positive ones).

The vast majority of prior research on parental monitoring of devices and online behaviors focuses on either young children using digital devices or older adolescents on social media. Early adolescence (e.g., middle school age range; 10–13 years) is a transformative transitional period between childhood and adolescence when the influence of family context and peer socialization begins to unfold parallel to early adoption of social technology (Marci et al., 2021). It is the period in which youth become smartphone owners, open social media accounts, and have online access at all times (Vogels et al., 2022). Yet less research on parental monitoring of media exists for this age group as compared to other ranges in childhood. This paper aims to expand our understanding of parental social technology monitoring behavior in the early adolescent years and its associations with problematic technology use leveraging a mixed-methods approach.

## Early Adolescent Technology Use and Monitoring

Online spaces offer young people both challenges and opportunities (Knorr, 2019). Yet, research often emphasizes the risks associated with adopting ubiquitous technology at younger ages. Some research has found increased smartphone use (i.e., persistent access to the internet) to be associated with negative feelings and behaviors in adolescence such as depressive symptoms, stress, anxiety, loneliness, and sleep disturbances (Charmaraman et al., 2021; Lee et al., 2014; Hawi & Samaha, 2017), while others have found little to no relationships between use and problematic outcomes (for full review, please see Odgers, Schueller, & Ito, 2020). Problematic internet use (PIU) is a construct that captures online and digital media use that is excessive, risky, or impulsive and that is related to adverse physical, social, or emotional impairment (Marci et al., 2021; Moreno et al., 2013). Most closely conceptualized as addictive behaviors related to self-regulation challenges (Moreno et al., 2013), adolescent PIU is associated with depression (Morgan & Cotten, 2003), problems with peer socializing (Shapira et al., 2000), loneliness (Cao & Su, 2007), and declining academic outcomes (Odaci & Kalkan, 2010). It is also associated with spending less time with family and friends, known as the displacement hypothesis (Chun et al., 2017), potentially creating tensions in parent-child and peer relationships (Valkenburg & Peter, 2007).

Parents’ concerns about persistent technology use often leads to monitoring strategies aimed at preventing risks and promoting healthier behaviors related to technology use (Beyens et al., 2022). Despite these monitoring efforts, PIU have yielded mixed results in relation to specific monitoring behaviors (Bleakley et al., 2016).

Adolescence is a period of increasing autonomy and teens may resist strict media rules, resulting in parent-adolescent conflicts and negative attitudes towards overly strict parents (Valkenburg et al., 2013). Researchers have argued that if parents fail to communicate their reasons behind screen rules and regulations, adolescents may rebel against this authority that counters their need for autonomy (Meeus et al., 2018). However, when they are explained, teens are more likely to respect parenting rules around media (Hiniker et al., 2016). Parental monitoring of media tends to vary with child age, as overseeing preschoolers’ game play on a tablet is different from helping a high schooler navigate Instagram (Davis, 2023). As the age of initiation to social technology decreases (Charmaraman et al., 2022b), parental monitoring must also adapt to understand and support younger adolescents’ use. A meta-analysis of parental monitoring found that much of the literature focuses on early childhood or older adolescents’ use, with no longitudinal studies covering the critical transitional period between childhood and adolescence (Collier et al., 2016). Since that review, (Richardson et al., 2021) investigated the impacts of technology use on sleep among young adolescents over time and found that parental control of screen time did not predict less use of technology or sleep quality over time. The research remains sparse for this critical period of development, yet young adolescents might be more vulnerable to PIU given that they are new adopters of social technologies while also undergoing significant psychosocial transitions (Marci et al., 2021) and learning to regulate thoughts and feelings (Silvers, 2022). Thus, little is still known about the relation between parental social technology monitoring and wellbeing outcomes in early adolescence. It is also worth noting that many of these studies have been conducted outside of the US, despite 95% of US adolescents reporting access to social technologies (Vogels et al., 2022).

## Parental Social Technology Monitoring Behaviors

Parental monitoring, in general, involves parents’ oversight of their children’s behaviors and whereabouts (i.e., surveillance), their rulemaking aimed at controlling children’s behavior (i.e., restrictions), and their active and open communication with their children (Stattin & Kerr, 2000). This framing has traditionally been called monitoring (e.g., Gentile et al., 2014; Wallace, 2021), but when studied in the context of media, it has more recently been coined mediation. This includes rules and limits of time or content known as *restrictive mediation* (Valkenburg et al., 1999) as well as efforts to promote critical thinking of the media by discussing central themes, character choices, and implicit messages of content known as *active mediation* (Austin, 1993). A final type of mediation of media is *deference mediation*, which is an intentional avoidance of restrictions, often in an attempt to showcase parental trust in children’s decision-making (Denham et al., 2000). Whereas restrictive and active mediation can be frequently found at all developmental stages, deference is more frequently studied in households with older adolescents (Padilla-Walker et al., 2010; 2018). Deference around technology monitoring is not entirely passive, with parents opting to engage in little to no restriction while prioritizing trust within the parent-child relationship (Padilla-Walker et al., 2012). While these forms of monitoring are often studied distinctively, it is important to consider how they may be used in overlapping, complementary, or interchangeable ways throughout this period of development. However, parents’ perceptions of the use of these differential strategies in such ways have not been as well studied during early adolescence. Parent involvement in adolescent social technology use has become integral to parenting in the 21st century. Therefore, triangulating the relations between parental monitoring strategies, family technology context, and parent’s perceptions of child’s technology behaviors is important.

Extant research across childhood ages finds that most parents do not use only one approach to media monitoring (Padilla-Walker et al., 2010) and much of the decision-making around media depends on the stage of development (e.g., early childhood, adolescence), child characteristics, and family norms. Similar to findings in other more traditional domains of parental monitoring (Stattin & Kerr, 2000), the utilization and impacts of various parental media monitoring remain mixed. For instance, among adolescents (12–17 years old) parents are more likely to communicate with daughters than sons about their social media use, yet parents are also less likely to intrusively monitor social media use of the opposite gender adolescent (Wallace, 2021). Though restrictive monitoring is viewed as protective against negative behaviors among children and adolescents, work by Sasson and Mesch (2014) show that restrictive monitoring can be associated with greater risky online behaviors. Furthermore, parents’ perceptions of youth’s ability to self-regulate their own media use is positively associated with low levels of active mediation strategies or high levels of deference (Padilla-Walker & Coyne, 2011). In their meta-analysis, Collier et al. (2016) reported that most studies do not distinguish between subtypes of parental mediation styles, and instead focus on the frequency of monitoring rather than the approach behind it. The differential use of *parental monitoring* and *parental mediation of media* in the extant literature, without clear articulation of their overlap, has limited our understanding of these types of parenting behaviors around media and how they are utilized with younger adolescents. Thus, for the purposes of this paper we ground these behaviors in the foundational parental monitoring literature that embodies the acts of media mediation and the relations of these strategies to family contexts and early adolescents’ online behaviors.

## Family Context in Monitoring of Adolescent Technology Use

Research on parental monitoring of social technology must consider the family ecosystems that influence technology use among developing adolescents (Jensen et al., 2021). Prior research finds parent-child relationship quality related to both parental monitoring strategies and child outcomes. For instance, teens’ secure attachment and positive relationships to their parents are associated with healthy social technology use (Badenes-Ribera et al., 2019; Davis & Koepke, 2016; Monacis et al., 2017) and positive parent-adolescent relationships can protect against developing PIU (Park et al., 2008). Parent attachment anxieties around relationships has also been found to be positively related to PIU among early adolescents (Marci et al., 2021). Conversely, negative, demanding, overprotective, and rejecting parenting styles have been identified as risk factors for PIU (Dogan et al., 2015; Park et al., 2008; Wu et al., 2016; Xiuqin et al., 2010).

Additionally, how parents use social technologies is also related to their young adolescents’ social media use (Hiniker et al., 2016; Reich et al., 2021) is an important part of the family context. *Technoference*, defined as the interruptions in social interactions or time spent together when a digital device is present, can be seen as part of the displacement hypothesis (Liu et al., 2020; McDaniel, 2015). These interruptions may occur during every day, face-to-face conversations such as playtime or meals. Studies have shown that parent digital media use is associated with the quantity (Radesky et al., 2015) and quality of parent-child interactions (Ochoa et al., 2021), such as lower responsivity (Hiniker et al., 2015), and hostility toward children who want attention (Radesky et al., 2014). In looking at family mealtimes as an important family routine, research finds both parent and child media use at the table to be associated with less conversation, less nutritious eating and lower parent-child interaction quality (Chitakunye and Takhar (2014); Linder et al., 2022; Van den Bulck & Eggermont, 2006; Yardi & Bruckman, 2011). Thus, both parents’ (technoference) and youth’s mealtime media use seem to contribute to displacement of parent-child interaction. Qualitative research has also demonstrated that both children and parents are uneasy with parental digital technology use during family routines: children believe parents need to be present and model good media habits (Hiniker et al., 2016) and parents feel less effective in their parenting when they are “multitasking” on a digital device (Radesky et al., 2016). A recent study found that parents’ perceived technoference in mother-child interactions was associated with parent-reported externalizing and internalizing child behaviors (McDaniel & Radesky, 2018). However, more research is needed to examine how these and other contextual factors relate to parental monitoring of media and parents’ perceptions of their child’s media use.

## Current Study

Given the importance of parental monitoring of media, the unique development capacities and online behaviors of young adolescents, the heterogeneity of family contexts, and the lack of research connecting parenting with early adolescent online behaviors, we explore the triangulation of how parents of young adolescents in the US monitor their middle-schoolers’ social technology use and how that is related to family contexts and parents’ perceptions of their child’s problematic internet use. Utilizing an explanatory sequential mixed methods approach (Creswell & Plano Clark, 2011) from a larger longitudinal study, parent surveys and semi-structured interviews target these three questions: (R1) *Are restrictive, active, and deference mediation associated with perceived PIU in early adolescence* (quantitative), (R2) A*re other family contexts (i.e., family closeness, mealtime use, parent problematic tech use) related to early adolescent PIU* (quantitative), and (R3) *How and why do parents use different monitoring strategies in settings of early adolescent technology use* (qualitative)? For the quantitative research questions (R1 and R2), we hypothesize:

H1: In considering early adolescents’ problematic internet use: (1a) Restrictive monitoring will be positively associated, (1b) Active monitoring will be negatively associated, (1c) Deference monitoring will be positively associated, and

H2: In considering the family context, (2a) family mealtime technology use will be positively associated with adolescent PIU (displacement), (2b) parents’ problematic technology use will be positively associated with early adolescent PIU (technoference), and (2c) family closeness and involvement will be negatively correlated to adolescent PIU.

Additionally, we explore how family context is associated with parental monitoring behaviors through this mixed methods approach. For the purposes of this study, we use parents’ reports on all survey measures related to their children’s PIU, family technology context, and parental monitoring strategies to provide a narrative from the parents’ perspective.

## Method

### Procedures

Drawing on an ongoing longitudinal study of middle school (6th - 8th grade) students’ technology use in multiple Northeast school districts, the current study focused on parents of the students from this larger study (Charmaraman et al., 2022a; Charmaraman et al., 2022b). The explanatory sequential mixed-method design (Creswell & Plano Clark, 2011) systematically integrates qualitative findings to further illuminate survey patterns of interest and potential mechanisms. After obtaining IRB approval from Brandeis University (on behalf of Wellesley College), study disclosures and Qualtrics survey links were distributed to parents at the middle schools through school-partnered contact lists, parent listservs, and e-newsletters from school administrative program coordinators in 2018–2019. All parents of middle school students were eligible to participate in a 20–30-min survey and entered into a raffle for multiple $25 gift cards. Surveys were available in English, Spanish, and Portuguese, with consent solicited on the first page.

At the completion of the survey, parents were asked if they were interested in a follow-up interview study. From this interview-interest list, we invited parents who reported both low and high monitoring practices. For summer 2018, we contacted 69 parents resulting in 18 interviews. For summer 2019, we contacted 68 parents resulting in 13 interviews. Study disclosures and signed consent were obtained prior to scheduling the interviews. Each participant received a $25 gift card for being interviewed.

Parent interview protocol. The semi-structured interview protocol asked parents about their digital media monitoring beliefs and practices; knowledge of their middle school child’s initiation of and current internet-based technology use, including those related to the Internet, smartphones, social media, and gaming; history of how they inform their monitoring styles, including advice given from external sources; and any approaches they use for dealing with problematic behaviors that are anticipated or currently experienced.

### Participants

#### Surveys

A total of 248 parents participated in the online survey and were asked to focus on their middle school child(ren) when responding to the questions. Twenty-one parents completed less than 20% and were removed, leaving an analytic sample of 227 with 90% identifying as the mother or female guardian of their child (*n* = 204) and 55% (124 parents) having a daughter. The majority identified as White (*n* = 165; 85.5%), followed by 7.8% identifying as Hispanic (*n* = 15), 4.1% as Asian American/Pacific Islander (*n* = 8), 2.1% Black (*n* = 4), and 0.5% as Multiracial (*n* = 1).

#### Interviews

In-person or phone interviews were done with 31 parents: 25 mothers and 6 fathers. We asked parents to focus their answers on their middle school child(ren). Exactly half of the parents referred to daughters (*n* = 15; 1 family had 2 daughters in middle school) and half referred to sons (*n* = 16) during their interviews. In terms of racial-ethnic composition, 20 parents were White (64.5%), 4 were Asian (12.9%), 3 Hispanic (9.7%), 2 Brazilian (6.5%), 1 Black (3.2%), and 1 Middle Eastern (3.2%). In order to understand variations among households, we purposefully over-recruited a more diverse qualitative sample in terms of gender and racial/ethnic background than the survey sample.

### Measures

#### Parental monitoring of adolescent technology use

First, parental level of *time restriction* of technology use was captured with a descriptive single item indicator “On a typical school day, what is the maximum amount of time you allow your child to go online or use the phone?” Responses ranged from 1–7; “less than 1 h” to “8 h or more” to “No Limit”. Then, three single item indicators from the Proactive Parenting Scale (Padilla-Walker & Coyne, 2011) measured *Restrictive* monitoring (“How often do you restrict your child’s internet use to avoid negative influences before they occur?”), *Active* monitoring (“How often do you help your child understand what he/she is seeing on the internet?”), and *Deference* monitoring (“How often do you allow your child to use the internet whenever he/she wants?”). Response options ranged from “1-Never” to “5-Always”.

#### Problematic digital technology use

Using three items from the abbreviated version (Moreno et al., 2016) of the Problematic and Risky Internet Use Screening Scale (Jelenchick et al., 2014) with two additional items, parents were asked to report their perception of their child’s problematic internet use (PIU) including when searching for information, playing online games, and using social media. This is a proxy measure for digital media consumption and items included, “How often does your child: lose motivation to do other things that need to get done because of the internet?, experience feelings of withdrawal from not using the internet?, feel nervous or anxious because s/he is away from the internet?, become moody or depressed when s/he is not online?, and lose sleep because s/he can’t quit what s/he is doing online?” Responses ranged from “1-Never” to “5-Always” with strong reliability (α = 0.88).

Parents were also asked about their own problematic device use (i.e., technoference) with the Problematic Digital Technology Use Scale (McDaniel & Radesky, 2018). Using a 5-point scale (1-*Never* to 5-*Very Often*), these 4 items had acceptable internal consistency (α = 0.79), with items such as: “When my mobile phone alerts me to indicate new messages, I cannot resist checking them.” and “I feel like I use my mobile phone too much.”

Lastly, family technology use during mealtimes (i.e., displacement of quality time) was assessed with two items: “While we ate together as a family, my child used a cell phone or tablet” and “I used my phone during meals while we ate together as a family (e.g., checking messages)”. Responses included a 5-point scale from “1-*Never*” to “5-*Always*.” The average of these two items had adequate internal consistency (α = 0.70).

#### Family closeness and involvement

Parents were asked to report on their relationship quality with their child with 4 items of parental knowledge, family closeness and involvement. The framing of each item assessed general family closeness such as “I get along very well with my child” and “I talked with my child about what is going on in his/her life”. Responses included a 5-point scale from “1-*Never*” to “5-*Always*” and the internal reliability of this measure was α = 0.64.

#### Parent and child demographics

Demographic characteristics included caregiver role (i.e., mother, father, guardian), parent’s race/ethnicity, parental education, employment status, and family income. Demographic information about the child included gender and school grade level (See Table 1). Parents were asked about their own and their child’s social media use, such as using platforms like Instagram, Snapchat, TikTok, etc. (Supplementary Fig. 1). Parents’ perceptions of why they believe their child joined social media was asked to contextualize the uses in early adolescence (see Supplementary Fig. 2 for detailed items of this measure).

**Table 1:** Survey parent and early adolescent demographics

	*Adolescent Gender*		
Variable	Boys (*n* = 103)	Girls (*n* = 124)	*Total (N* = 227)
*Parent Reporter*			
Mother/Female Guardians	87	117	204
Fathers/Male Guardians	16	7	23
*Child School Grade*			
6th Grade	45	51	96
7th Grade	31	40	71
8th Grade	27	28	55
*Household Income*			
<$35 K	5	6	11
$35 K–$50 K	4	7	11
$50 K–$75 K	6	7	13
$75 K–$100 K	12	11	23
>$100 K	42	55	97
Did not disclose	20	19	39
*Marital Status*			
Married	70	91	161
Separated	4	2	6
Divorced	11	3	14
Widowed	1	1	2
Never Married	4	9	13
*Parent Education Level*			
Some/HS Graduate	12	16	28
Some/College Graduate	41	46	87
Graduate/Professional School	38	44	82
*Employment Status*			
Full-Time	59	69	128
Part-Time	13	24	37
Looking for Work	1	2	3
Stay at home caregiver	15	10	25
Unable to work	2	0	2
Other	0	1	1

### Data Analysis

#### Quantitative

Survey data were analyzed using R 4.1.0 (R Core Team, 2020). Descriptive statistics of the sample demographics and supplemental materials on parents’ understanding of technology use were included in the current study. To address H1 and H2, bivariate correlations were conducted as initial descriptors between parental monitoring, family contexts, and technoference on early adolescents’ problematic use. Then, a multiple linear regression model was performed by to assess the magnitude of associations of specific indicators of children’s problematic technology use by parental monitoring and family technology context (i.e., family closeness, technoference for parent and child) regressed together in a singular model. Covariates included caregiver roles (i.e., mother, father, etc.), race/ethnicity, household income, employment status, marital status, parent education, child’s gender, and grade in school.

#### Qualitative

Recorded interviews were transcribed verbatim, verified by the research team, and imported into NVivo software (NVivo 12 Version: Plus). An initial open coding phase was used to categorize, sort and code, which was conducted separately by four undergraduate research assistants trained in social media research and qualitative coding procedures. Initial nodes were created a priori according to the main interview questions. New nodes and sub-nodes were then created through both deductive and inductive processes. The second author then proceeded to develop an electronic memo trail. This was written during the primary and secondary coding phases to keep track of first impressions, reactions to the interview content, developing ideas, and emerging themes. This allowed for similarities and patterns to emerge and be recorded for the purpose of theme development. The second and third authors both conducted axial coding (Strauss, 1998) which involved continuous cross-checking, refining, and analysis to confirm that the themes and subthemes were categorized and defined accurately, and to classify higher order themes that provided meaning to connect lower-level codes. The reliability agreement between primary and secondary coders was at 93.6%.

#### Mixed methods

Using an explanatory sequential QUAN → qual mixed methods design (Creswell & Plano Clark, 2011), the quantitative analysis guided purposeful sampling of parents invited for the qualitative phase, and the qualitative analysis of parental monitoring strategies were partly based on groupings extracted from the quantitative phase. That is, the robust results between PIU and restrictive monitoring (Table 3) rationalized our expansion of coding categories within restrictive monitoring in the qualitative analysis. To complement the limitations in quantitatively analyzing emerging and less frequent types of monitoring behaviors, the qualitative exploration allowed us to explore other monitoring behaviors more deeply. Thus, the points of interface were at different time points of the study including data collection, data analysis, and interpretation. Analysis of qualitative data refines and explains the nuances of the statistical results by exploring parents’ views in more depth (Tashakkori & Teddlie, 1998).

## Results

### Quantitative Findings

#### Descriptive statistics

Survey sample demographics can be found in Table 1. Additionally, supplemental descriptors were analyzed to contextualize the understanding of technology use by caregivers. On average, parents were active users of social media with only 5 (2.2%) not using any form of social media (Fig. S1). Many parents (41.9%) believed their child adopted social technologies to connect and share things with friends, thus characterizing the importance of online social connectedness (Fig. S2). On average, parents used a mixture of parental monitoring strategies as shown on Fig. S3b. Across the strategies, active monitoring was the most endorsed with 62.9% of parents expressing they use this strategy “often” or “always.” However, the majority of parents (84.9%) endorsed placing time restrictions on technology use, ranging from 1 to 6-h limits (Fig. S3a) and 60.4% of parents acknowledged that they knew when their early adolescent was online or using the internet (Fig. S4).

#### Parental monitoring and adolescent PIU

Among the three types of parental monitoring reported in this study (Table 2), restrictive monitoring only was significantly and positively associated with children’s PIU (*r* = 0.19, *p* < 0.01). Active and deference monitoring were not significantly correlated with adolescent’s PIU, which contradicts H1b and H1c. Furthermore, active monitoring was positively associated with restrictive monitoring which may be indicative of the fluidity of monitoring behaviors. Deference and restrictive monitoring were negatively associated with each other; however, these monitoring behaviors were both positively associated with technology interference during mealtimes (*r*_deference_ = 0.17, *p* < 0.01; *r*_restrictive_ = 0.17, *p* < 0.05). Children’s PIU was also associated with tech interference during mealtimes (*r* = 0.18, *p* < 0.01).

**Table 2:** Survey bivariate correlations

*Variable*	1	2	3	4	5	6	7
1. Restrictive Parental Monitoring	-						
2. Active Parental Monitoring	0.21^**^	-					
3. Deference Parental Monitoring	−0.25^**^	-0.06	-				
4. Child problematic internet use	0.19^**^	-0.05	-0.06	-			
5. Parent problematic phone use	0.08	-0.05	-0.05	0.23^***^	-		
6. Family closeness and involvement	0.08	0.28^***^	0.01	−0.43^***^	-0.13	-	
7. Family meals tech interference	0.17^*^	0.02	0.17^**^	0.18^**^	0.30^***^	-0.12	-
**Mean** **(SD)**	2.71 (1.11)	3.83 (0.94)	2.70 (1.14)	2.07 (0.75)	2.42 (0.79)	4.50 (0.42)	1.84 (1.75)
***N***	221	221	220	216	214	224	224

^*^p < 0.05, ** *p* < 0.01, *** *p* < 0.001; significant results indicated in bold

#### Multiple regression model

To further test and identify unique indicators of adolescent PIU, a multiple regression model controlling for relevant covariates was conducted (Table 3). For monitoring behaviors, restrictive monitoring remained significantly positively associated with adolescent’s PIU (*β* = 0.17, *p* < 0.05) when accounting for variation contributed to the model by the other variables of interest (H1a) and active and deference monitoring remained non-significant. Above and beyond the other indicators in the model, family closeness and involvement showed the strongest magnitude of negative association to adolescent’s PIU (*β* = 0.41, *p* < 0.001), and parent’s own problematic phone use was a positive indicator of children’s PIU when accounting for covariates and monitoring behaviors (*β* = 0.15, *p* < 0.05). The mealtime technoference became a non-significant indicator when accounting for the variable of interest in the model.

**Table 3:** Multiple regression model parental monitoring on early adolescent problematic internet use and family context indicators

	Child’s Problematic Internet Use			
	*b*	SE	*ß*	*p*
*Covariates*				
Child grade level in school (age)	0.03	0.06	0.04	0.61
Child gender	0.21^*^	**0.10**	**0.14**	**0.04**
Family income	−0.02	0.03	−0.07	0.36
Parent relationship	0.06	0.18	0.02	0.74
Parent employment	−0.03	0.04	−0.04	0.53
Parent marital status	0.05	0.06	0.06	0.42
Parent education	−0.05	0.08	−0.05	0.52
Child race-ethnicity	−0.09	0.06	−0.10	0.19
*Family Variables*				
Mealtime technoference (parent and child)	0.06	0.07	0.06	0.41
Family closeness and involvement	−0.71^***^	**0.12**	**−0.41**	**<0.001**
Parent problematic phone use	0.15^*^	**0.07**	**0.16**	**0.03**
*Parental Monitoring Behaviors*				
Restrictive Monitoring	0.12^*^	**0.05**	**0.17**	**0.02**
Active Monitoring	0.04	0.06	−0.05	0.49
Deference Monitoring	−0.03	0.05	−0.05	0.55
*F*	4.78			
*Adj. R*^*2*^	0.23			
*n*	181			

Statistically significant results are indicated in bold. Beta (*ß*) indicates standardized regression coefficients. Covariates included child grade level in school as a proxy for age, gender, parental relationship to child, child race-ethnicity, family income, parent employment status, parent marital status, and parent highest education level

* *p* < 0.05, ** *p* < 0.01, *** *p* < 0.001

### Qualitative Findings

Across the 31 interviews, we deductively coded under the themes of *how* parents used restrictive, active, and deference monitoring approaches, and *why* they used the approaches they described. A fourth monitoring approach of surveillance emerged and was coded. In addition, a theme emerged related to the uncertainties parents felt about the approaches they have chosen to use.

#### Theme 1: How parents monitor social technology use (Table 4)

Restrictive monitoring was the most frequently described strategy across all interviews, used by all but one parent (n = 30). Of the 495 parental monitoring thematic references, 257 were restrictive, 111 active, 63 deference, and 64 surveillant. The most common restrictive approaches were control of device settings (“I restricted the internet …so she can [only] go to certain websites [that] I put in”) and withholding devices (“The deal was that he follows the rules or he loses the phone.”). Other techniques included the use of passwords, requiring chores or other duties to be completed to earn use of the device, and setting limits on time, contacts, and locations of use. A mother pointed out: “When he’s done with his homework, I shut down the internet on that device… and then the computer or the Chromebook physically reside in my room.”

**Table 4:** How and why parents use specific monitoring approaches

Monitoring Strategies	Illustrative Quotes
Restrictive Strategies	
*Password*	“…whenever he would use the iPad we had to log into the iPad with the password and let him play.”
*Control over settings*	“I grab the tablet and say, ‘Let me change the settings here and up the security.’”
*Parental control systems*	“I put a lot of…restrictions where in order for them to get an app, they always have to check with me…Family Sharing”
*Don’t give/take away device*	“He is probably one of the few kids who doesn’t even have his own phone. So, he doesn’t use any social media.”
	“Found a few songs that had the F-word in it, so we took the phone away for… a week or two.”
*Time limits*	“When he’s on electronics, it’s limited to the weekends and he’s not allowed to play more than 2–3 h a day.”
	“I limit the time he can do this game, like 30 min at a time, or if more than two friends are here, they can use it for 60 min.”
*Contact limits*	“The first rule is that he can’t play with people that he doesn’t know.”
	“(I) make sure he only plays with his approved friends, and I check their username addresses.”
*Location limits*	“No devices are allowed in the bedroom. No devices when they’re eating.”
	“Computers are always at the kitchen table. And the phones are either in the kitchen or in the dining area.”
*Requiring chore completion*	“He has various responsibilities that he has to do at home before he can even access any electronics.”
Active Strategies	
*Give examples from real life*	“We talk about college coaches since she likes to play sports all the time. We talk about when she goes on job interviews someday that this is what they look at. This is the first thing they go through.”
*Play/use together*	“[My husband] plays the games with my son.”
*Try to set good example*	“A lot of the parenting we do, we try to set a good example. And so, they know I’m on Facebook, they know I post stuff, and if I want to post something about them, I will ask them for permission.”
*Empathize*	“So we’ve talked a lot about that…, ‘Oh yeah, I feel the same way’. I’ve sort of talked to them almost like a friend. Like, ‘oh, I feel like that too. I’ve been through that.’ So we sort of talk that out.”
*Ask questions*	“We try to reflect back to her and say ‘What do you think? You’ve made a lot of good decisions. We’re really proud of the good decision you’ve made. What do you think you should do?’…We haven’t said, ‘I ban you or restrict you from talking to this person.’ We might say, ‘Well, geez, if you’re having these negative exchanges with this one friend of yours, is it worthwhile? Do you think may be you want to not communicate with them for a while, or block their messages?’”
*Establish clear expectations with no threat of punishment*	“We can pick up [the phones] at any time and look at them. And there should be nothing on the phone that we would be surprised to see. That’s our expectation… It’s really about creating an expectation about ‘this is how we expect you to use it.”
*Be conversational and not prescriptive*	“I try to be more conversational with them instead of telling them what to do. Like, we’ve talked a lot about being on Instagram and seeing people at a party and realizing that you weren’t invited to that party and how that makes you feel.”
Deference Strategies	
*Don’t friend online*	“I always assume that kids wouldn’t want me to be their friend on social media.”
*Don’t look at devices*	“I’ve never really been that obsessive…. I look more for the signs of his behavior than looking into his devices.”
*Don’t establish rules*	“He spends more time online than I would like. At the same, he’s getting things done. The school projects or homework get done and he’s getting reasonable grades. He never misses homework. He’s doing fine in school. So my rule is as long as he’s doing all that, he’s pretty much free…. We’re pretty, I would say, soft on the rules.”
Surveillant Strategies	
*Check browser history*	“I frequently check his browser history. I’m constantly checking it so he knows he’s being tracked.”
*Watch while online*	“When they are using their devices, I will pop my head in and see what’s on the screen….fairly regularly.”
*Follow on social media*	“My older kids keep an eye on her on SnapChat, and I keep an eye on her on Instagram.”
*Verbal check-in*	“If they’re texting or looking something up on their phone, we expect them to ask us.”
Reasons for Use	Illustrative Quotes
Restrictive	
*Addictive*	“… these games are addictive…” “…it’s like a drug to them.”
*Displacement*	“There are other experiences in life besides looking at a screen and if you’re always looking at a screen you won’t know what those other experiences are.”
*Child not mature enough*	“We just felt that he was not mature enough to understand that this is just a game that you do occasionally…”
*Content not appropriate*	“We took away a game… because it was too violent.”
	“…the pornography stuff is really the most terrifying; I just think it’s so damaging to children.”
*Online contacts*	“I was concerned about who they were contacting online. Because you could speak to strangers, you could speak to anybody.”
*Impact on brain*	“We don’t allow our kids to go on all different types of TV shows. I think it’s mind-boggling at that age. It’s like rotting your mind or something. It is so much. They go in and they process it…with their brains.”
Active	
*Child mature enough*	“I think she’s at the age where I would almost want her to start dipping her toe into this world a little bit. In a controlled way. Because I want her to learn how to do it when it’s still low risk.”
*Stay up to date on trends*	“We’ve tried to keep up on the trends and tried to read about different options and stuff, so we’ve talked to him about social media.”
*Too restrictive doesn’t work or can backfire*	“I’ve seen what taking away the phone does. I think it just pushes the kid to be more sneaky … He’s really clever”
	“You can put as much control on a person as you like, but one day that person will break. And I think that’s what’s happening with the kids. We’re trying to control them more and more… overloaded with restrictions… they start fighting back. “
*Prefer that info comes from parents and not others*	“You have to have these conversations with them. This is reality right now. They have access to anything and everything that they want, and sometimes that stuff is true, and sometimes it isn’t, but either way, it’s out there. They’re going to hear about it and I want her to have the right information, and not the false information. So we’ve had a lot of conversations that we may not have had if there wasn’t the social media, internet, phone.”
*Parents’ role is to provide tools for life*	“So I have to make it an open conversation, and have trust, and just give her whatever information I have… Hopefully she’ll take the tools that I gave her, and listen to them in the back of her head before she sends a picture.”
*Helps parent and child become closer*	“This is how I found to be close with him, I listen to music that he likes, when we are in the car together, we put on his Spotify, and I think it’s a good way for me to connect with him. So I sacrifice myself sometimes…. I try to be a friend of him in his Instagram and Snapchat…. I cannot fight against this so let’s play this together.”
	“I tend to be more liberal and people sometimes think that I don’t care but I think it’s important for us to see how we can use social media to be close to them.”
*Fits into global parenting strategy*	“It’s just the strategy I use with pretty much all my parenting which is, ‘tell me where you’re coming from’, just talking it out, explaining to her what my rationale is, giving her a lot of space to explain to me so that I can understand her rationale. “
Deference	
*So child can fit in*	“I’ve never discouraged him to use it because I feel like if everybody’s doing it, I don’t want him to not use it.”
*To give child responsibility and opportunity to learn*	“I try to allow him to take the responsibility to stop the gaming. Sometimes I say… I trust that you’re going to end this at 11.”
	“I will always step in on your behalf, if you want me to, but it is really important for you to learn peer to peer relationships. But we are always here as your backup, and if you need us, we will come.”
*To give child privacy*	“He has a right to privacy. And so, I have not looked in his phone. I know some of my mom friends have. I don’t really feel comfortable doing it and I think they are kind of comfortable with the idea.”
*Child is meeting all expectations (school, etc..)*	“I don’t feel the need [to have conversations about social media use] because she’s not using or abusing it. But if I do, I will have… Currently she’s fine.”
*Technology offers benefits*	“He watches a lot of YouTube stuff and I would say about one-third of those are kind of educational which is good. He does learn a lot from online sources.”
*Parent feels unable to engage child or to compete with external influences*	“Every time I try to get him involved in a conversation [about social media] it’s something he has heard so much about and he thinks he knows all the rules…. and the ramifications of getting online. He seems to know all that, so he usually refuses to continue the conversation.”
	“There’s too many things that can influence them in a lot more powerful way. The influence from parents is very limited.”
*Parent is not tech savvy*	“I don’t have access to these accounts. I don’t know exactly what’s in there… I think I should. I should try to get a Snapchat account and try to link with him just to know what’s going on…. Snapchat is just not something that I personally use at all. So it’s just to get account to spy on him is something that I should have done, but I just haven’t done yet”.
*Parent trusts child*	“I hope I’m not wrong, but they’re good kids. I sort of trust them to make good decisions.”
	“I cannot believe how many of our friends track their kids with the GPS things. I don’t do that. I feel very mixed about that because we did not have that as kids, and I do think that it really breaches a trust thing.”
*Parent doesn’t want to shield child*	“It’s life and I can’t shield her… I don’t want her to feel sad or left out. She has a full busy life all on her own. She doesn’t need to be feeling like she’s missing something.”
*Parent permits child to emulate parent*	“I’m a social worker, so I work with a lot of kids who have addiction to social media or a cell phone, and their parents are just as bad as they are. So it’s really difficult to tell them to have their kids not do it when they themselves are in the throes of addiction to the same thing.”
*Child is not active user*	“She never used it, so we don’t really have rules except like late at night, she’s not allowed to use it.”
*Monitoring not priority*	“I think I would like to get more access to his social media account or something. Which is something I’ve been meaning to do.”
*Too difficult on parents*	“We have friends who monitor every message their kids send…I don’t want to spend an hour and a half doing that at night. Partly because I feel like you have to trust your kid and this is part of the world we live in, but the constant vigilance is exhausting.”
*Parent doesn’t want to be overbearing*	“I have not stepped in, and I have a friend who just recently stepped in, and it ended up worse for her daughter, which doesn’t surprise me. Go talk to the kids at the school playground, even when they’re little. Being an overbearing mom is not a good thing for your kid.”
*Parent doesn’t have concerns*	“Her social network behavior… was never a concern of mine. She knows what to do…. may be because she heard us talking with her older siblings and… she is a smart girl.”
Surveillant	
*Fear of inappropriate content*	“I take a look… to reassure myself that she’s not posting stuff that she may not even be aware that is problematic….”
*To limit time spent online*	“I think we’re … kind of checking in with him… he’s more likely to be that kid who would sit in the same place for 12 h and never let the phone leave his hand…”
*To track whereabouts*	“I could literally… LoJack her… see where she was with the “Find my iPhone” app and I could reach her at all times.”
*To gain insight into private life*	“The way he posts a picture and how his friends react to his post, I kind of see that he’s a character, his role or position in the school, and that’s giving some insights from a different angle.”

Engaging in dialog was the most commonly reported active monitoring technique – a general strategy mentioned by 23 parents. This included asking a lot of questions, and encouraging the child to ask questions too, so that discussions were bidirectional and not prescriptive. As a mother described: “I try to take more of the approach of ‘let’s discuss about why’.” Many parents also tried to caution children about consequences of using social technology: “I would tell [my child] that the HR from the college would be checking our social media to look at our behavior or what we post.” Other active approaches included using technology together, modeling the behaviors they wanted to see, and establishing clear expectations without the threat of punishment or loss of privileges. As a parent pointed out: “We can pick up [children’s phones] at any time and look at them. And there should be nothing that we would be surprised to see. That’s our expectation. We didn’t make it a punitive issue, like no cell phone for a week. It’s really about creating an expectation ‘this is how we expect you to use it.’”

Fifteen parents mentioned deference monitoring strategies, mostly defined by what parents chose not to do, such as not friending their child on social media or looking through their devices: “I look more for the signs of his behavior than looking into his devices.” Deference practices included being generally “soft on the rules,” as one parent admitted, and some parents simply said they did not have rules at all.

Although not measured as part of the quantitative survey, surveillance was a frequently mentioned monitoring practice in the interviews. The most common surveillant method involved periodically checking emails, texts, social media accounts and browsing history. Some parents acknowledged watching the child from a distance (“When they are using their devices, I will pop my head in and see what’s on the screen.”) or following their child on social media silently, as compared to the active strategy of requesting to be the child’s online friend in order to openly discuss social media content. Such use for some was done without the child being aware of the monitoring: “Over the years I randomly grab their phones and I check, I try to do it at night so they don’t know I’m doing it.” Other children were warned in advance: “I frequently check his browser history. I’m constantly checking it so he knows he’s being tracked.” Checking behaviors were described by 27 parents at different frequencies from “every couple of days” to “couple of times a week” to “constantly checking to see what’s getting posted or who’s posting.”

Most parents reported using more than one type of monitoring approach and many described using all three at different times. For instance, one mother had an app that allowed her to turn off her son’s devices at any time. She also had very rigid rules around the times that he was allowed to use them. But she engaged in frequent conversations with him about how to use them responsibly: “I’ve never discouraged him to use [the device] because I feel like if everybody’s doing it, I don’t want him to not use it. I’d rather he use it responsibly…So, I think that’s been kind of more of the conversations that we’ve had.” And she even showed some deference when she said, “I don’t really delve into exactly what he is doing” and “I always assume that kids wouldn’t want me to be their friend on social media” and “I say to him ‘I trust that you are going to end this [device use] at 11 pm.’”

#### Theme 2: Why parents use specific monitoring approaches (Table 4)

Parents described a variety of reasons for using their chosen monitoring strategies.

#### Restriction for intervention versus prevention

While a large number of parents used restrictive practices, their reasons for doing so clustered into mainly three categories: concern that social technologies are addictive or could replace other healthier activities; a belief that children are too immature to be exposed to inappropriate content or online strangers; and a fear that online activities can negatively impact the brain.

Parents typically chose to restrict their child’s online activities either as a reactive (i.e., intervention) or pre-emptive (i.e., prevention) approach. About a third of parents expressed concerns about problematic internet behaviors (e.g., ignoring parent while playing games) which often prompted them to use restrictive strategies to curtail the behavior (e.g., no free access to Internet with the goal of limiting game use). A parent who was concerned about falling grades felt that intervening by taking the phone away made a difference: “So when I took that away I noticed the teacher was like his grades are improving, and so that’s what I have to do to get your grades up.”

Other parents described using restrictions as preventive measures. Their views largely stemmed from concerns around technology harms: “I was an engineer in Silicon Valley, I know what this technology does. Steve Jobs is like ‘I would never give my kids an iPad’ and I can see it for what it is, and a lot of people can’t.” Some of these concerns arose from previous difficult experiences with older children and even spouses: “I watched my son when he first started doing video games and Minecraft… he has an addictive personality, and I watched my husband’s use and I thought this is stealing kids’ childhoods.” Parents also reported restricting to prevent negative outcomes for children that were not yet mature enough for independent use: “At this moment I can’t trust him. He’s a good kid, but he can’t think of the result of what he is doing on social media. But I don’t think I can supervise him. So that’s why I don’t want him to have an account at this moment… I just don’t know how to supervise.”

#### Fears fueling restrictions

The potential to meet strangers online was a commonly reported reason for restrictive practices: “I don’t really let him play this game because…I think he can fight against strangers.” Some parents felt their child was too “innocent” and “naive”, and one parent expressed concern about her daughter being “blackmailed,” or having her “identity taken or things found out about them.” Other fears that parents shared included the impact of inappropriate content (e.g., violence, pornography) and the potential physiologic effect of technology on the brain: “In terms of neuroscience, like what it does to your brain…I have this vague understanding that looking at a screen and constantly being distracted and your thoughts moving from this to there is probably not great for neural pathways.”

#### Choosing active out of concern that restrictive won’t work

Parents using active approaches expressed concerns that being too restrictive could backfire, especially when they perceived their child to be at an appropriate and responsible age. “I’ve seen what taking away the phone does. I think it just pushes the kid to be more sneaky.” Another parent noted, “If I were to be strict-strict, I think she wouldn’t have it.”

#### Active as part of a global parenting strategy

Many parents felt their job was to provide their child with tools for life. Thus, they engaged in active discussions with the goal of having the child self-reflect and make their own decisions: “So I have to make it an open conversation, and have trust, and just give her whatever information I have to protect herself, and then inevitably, she’s going to do what she wants to do. Hopefully she’ll take the tools that I gave her, and listen to them in the back of her head before she sends a picture.” For others, collaborative discussions simply fit within their global parenting strategy: “It’s just the strategy I use with pretty much all my parenting which is, tell me where you’re coming from; just talking it out, explaining to her what my rationale is, giving her a lot of space to explain to me so that I can understand her rationale.” Active monitoring also helped to reassure parents that information about the world came from them rather than peers or other potentially untrustworthy sources: “They’re going to hear about it and I want them to have the right information, and not the false information. So, we’ve had a lot of conversations that we may not have had if there wasn’t the social media, Internet, phone.”

#### Active monitoring to remain close to the child

Some parents felt that active monitoring helped them feel closer to their child: “If social media is so important in their lives, as parents, how can we learn and take advantage of that to be closer to them?” Moreover, some parents admitted that discussing media with their child was a way for parents to keep up to date on trending topics that are popular with the next generation: “We’ve tried to keep up on the trends and tried to read about different options and stuff, so we’ve definitely talked to him about social media.”

#### Deference approaches that reflect on the child

While deference practices were the least common, reported by less than half of the parents, the reasons offered for why a parent might choose to be deferent were diverse, and included both child-focused and parent-focused rationales. As opposed to restrictions which were applied to manage present or future problematic behaviors, a hands-off approach was more common in families where the child was meeting expectations at school and home and where the child was deemed to be trustworthy. Parents often wanted their child to fit in with other tech-using children: “I’ve never discouraged him to use it because I feel like if everybody’s doing it, I don’t want him to not use it.” Some of these parents felt that restricting could unnecessarily shield a child from the world, and from the many benefits that social technology can provide: “I try not to confront him unless he does anything bad online which has really not been the case, as far as I can tell. …He watches a lot of YouTube stuff and I would say about one-third of those are kind of educational, which is good. He does learn a lot from online sources.” Some parents expressed the opinion that deference bestowed upon a child a sense of responsibility and an opportunity to learn in a setting of privacy and trust: “He’s 14, so I feel he has a right to privacy. And so, I have not looked in his phone. I know some of my mom friends have. I don’t really feel comfortable doing it.” A few parents did not feel the need to restrict or engage in active monitoring when their child was not an avid user of social technology: “She never used it, so we don’t really have rules.”

#### Deference approaches that reflect on the parent

Parent-centered reasons for remaining deferent included a wish to not be perceived as overbearing; a lack of confidence in their own ability to oversee device use (e.g., not tech-savvy) or to manage their children (e.g., helpless as a parent, child not cooperative); and an acknowledgement that they too might have problematic internet behaviors and would feel hypocritical for expecting their child to behave otherwise: “I can’t tell them they can’t have their iPads at the table if I’m sitting there, you know, checking emails for work or doing whatever.” Some parents might not have actively chosen a deference path but found it to be a default state if they had not made efforts to restrict or actively engage: “I think I would like to get more access to his social media account or something. Which is something I’ve been meaning to do.”

#### Surveillance before other monitoring approaches, or as an isolated strategy

Surveillant strategies were often a preliminary step before engaging in restrictive or active monitoring. Parents reported surveilling a child’s online activities out of fear of finding “problematic stuff,” suspicious contacts, or addictive behaviors, which might ultimately result in consequences: “My wife and I [said] to him, ‘We’re gonna check your phone and make sure you’re not downloading the songs that has F-words in it’, and we actually one time found a few songs that had the F-word in it, so we took the phone away for a week or two.”

In contrast, other parents engaged in surveillance without a subsequent restrictive or active strategy. For example, some parents felt that simply being watched might keep the child in line: “I’m constantly checking it so he knows he’s being tracked.” Surveillant practices were also used for reassurance, such as knowing a child’s physical location, or gaining insight into their child’s plans and experiences: “Honestly, the main reason I check it when I do is because something’s come up where she’s making plans with a friend, and she’s doing it herself, and I’m frustrated because I can’t get her to figure out the plan, if it involves me driving, or whatever.”

#### Theme 3: Uncertainty in monitoring approaches

Regardless of approach, many parents expressed uncertainty that they were doing the right thing for their child and felt the need for external or peer validation. There were many examples of parents feeling alone in their decisions. One self-professed “super strict” mother would have appreciated more scientific backing for the validity of her choices: “I would love some cold, hard facts stating that yes, there are negative social, emotional impacts to kids by being on this stuff. I would like validation for my restrictiveness —I feel like I’m the only parent in [town] who doesn’t let my 13-year-old have a smartphone. But yeah, I would love some validation that I’m doing the right thing because it’s hard.” Parents also expressed ambivalence and a sense of being conflicted, especially when they noted other families taking different approaches: “I have two different feelings. I don’t want him to have access, but all of his friends have access to the Internet, which means they may have access to social media, and he might be left behind.” Some wavered in their intent to check on their child’s social media accounts: “I should try to get a Snapchat account and try to link with him just to know what’s going on…to get an account to spy on him is something that I should have done, but I just haven’t done yet.” Other parents wanted to respect their child’s privacy (e.g., “It’s invasive - a private space”) but were unsure about the appropriate timing or parameters for restrictions: “You don’t want to be super police…It’s hard to know exactly what the right age is.”

Some parents who reported using deference approaches seemed to feel guilty with elements of self-questioning. For instance, “No, I don’t [have specific rules], and I probably should put some.” Another parent questioned herself, “Would she get pulled in on some meanness and be complicit with it? I’m not worried a lot about sexual exploitation or grooming. I’m not worried about that, may be I should be?” Parents also felt embarrassed about not monitoring: “[It’s] almost embarrassing to say out loud [what games he plays]. He’s ten and, like, there’s almost nothing we won’t let him play.”

Parents who reported mainly using active communication strategies seemed to feel less conflicted and a bit more confident that everything would work out as compared to those who used mainly restrictive or deference approaches: “You’re just trying to do the best you can and hope that you’re doing the right things because there’s no immediate feedback that it’s going perfectly swimmingly well.”

## Discussion

This mixed-method study assessed different types of parental technology monitoring strategies and how family contexts relate to young adolescents’ problematic internet use. In line with previous work that demonstrates that parents use a variety of monitoring behaviors (Padilla-Walker et al., 2010), our participants similarly reported a variety of approaches. Restrictive monitoring was associated with greater children’s PIU, which aligns with prior research (Sasson & Mesch, 2014) providing support for hypothesis 1a. However, active and deference monitoring were unrelated to children’s PIU (not supporting hypothesis 1b and 1c). Our discussions with parents help shed light on these findings by describing the variety of ways that these strategies are applied and a range of reasons why. Parents often described restrictive practices being utilized as a form of intervention. Thus, the youth that are engaging in problematic behaviors may be more likely to experience greater parental restrictions. Conversely, parents who described their child as more responsible and valued building capacity for healthy online use supported more active strategies. Such patterns suggest a reciprocal relationship between parental monitoring strategies and children’s online behaviors (Nikken de Graaf, 2013), much like other domains of parenting (Bell, 1979).

Family context also played a significant role in parental monitoring and young adolescents’ online behaviors (Hypothesis 2). Although parents’ monitoring behaviors were not related to their own problematic device use, parents’ self-report of their problematic phone use and family technoference during mealtime were positively related to parents’ perception of their child’s PIU (Table 2). Such findings might indicate a displacement of higher quality parent-child interactions through device use (Valkenburg & Peter, 2007; McDaniel, 2015). This association also highlights the consistent positive relation between technoference and parents’ own problematic use of their device, which is a significant indicator of the family influence. It might also represent a form of socialization, in which parents’ model behaviors that their child emulates (Rogoff, 2003). Furthermore, our results show that displacement during mealtime was strongly associated with deference monitoring. This pattern was shared during the interviews with some parents who use deference strategies feeling hypocritical if they required standards for their child that they did not abide by themselves.

Our qualitative findings also demonstrated that parents used rules and restrictions for both intervention and prevention, often out of fear (inappropriate content, strangers) derived from personal experience with older siblings or spouses. The three primary categories for parental social technology restrictions were (a) a concern that social technologies are addictive or displace healthier activities, (b) avoiding exposure to inappropriate content or online strangers, (c) a fear that screens can negatively impact the brain. When describing the reasons for choosing specific strategies, parents provided these same few reasons for restrictive strategies. In contrast, deference was the least mentioned strategy, but parents provided much more detail and diversity of reasons for using this approach, perhaps to justify an unpopular and often misunderstood approach amongst their parenting community. Extant research supports deference as facilitating autonomy and trust for older adolescents (Padilla-Walker et al., 2018), but our data indicates that parents of younger adolescents may be willing to let go of restrictions if they feel it is warranted or simply hopeless. Importantly, parents’ strategies that focused on trust and experience rather than specific rules or restrictions would be missed in common parental mediation of media measurement that that focus on the presence or absence of rules and restrictions only (e.g., Nikken & Jansz, 2014; Ho et al., 2020; Beyens & Valkenburg, 2019).

Overall, our qualitative findings revealed that many parents were not confident in their monitoring choices. Some avoided restrictive approaches for fear that they might be ineffective or even backfire, which some research has found (Nikken de Graaf, 2013). Other parents wanted more information or validation of how best to parent around technology. We found surveillant monitoring to be a popular way to track usage, uncoupled from concrete rules or regulations of use. Surveillant behaviors were not communicative or “active” strategies, and often involved a mistrust of their child (or child’s online networks), which is counter to deference approaches that believe in children’s right to privacy and a trust that they will do the right thing. Surveillance is a common strategy in parental monitoring research (Stattin & Kerr, 2000), but less often considered in monitoring of media use specifically.

In surveys and interviews, we found that there was no one-size-fits-all approach to parenting around technology. Some parents combined restrictive and deference approaches, either at different times of day (e.g., phone stays in the kitchen overnight, but there are no time limits during daytime hours) or on different devices (e.g., Net Nanny installed to control movie watching, but no phone rules are established). Research on parental monitoring around media often assesses practices as ubiquitous across devices, uses, and platforms. Our findings suggest that a more nuanced exploration into parenting around media is needed.

Parents are active social technology users (Pew Research Center, 2015). In our sample, almost all parents used social media and many expressed challenges regulating their own use. Such technoference was related to children’s problematic behaviors and parents’ perception of family closeness. Still, many parents described using monitoring strategies that were based on knowing their child well. Parents utilizing active monitoring strategies valued bidirectional conversations, more frequent communication about boundaries, spending more time together, and not using threats or punishment to motivate healthy social technology use. Research on active monitoring strategies consistently finds that open communication facilitates trust and is both protective against detrimental child behaviors and promotive of positive outcomes (e.g., Kerr et al., 2010; Crouter et al., 1990, Stattin & Kerr, 2000). Thus, it is not surprising that such parent-child communication behaviors are associated with less problematic online behaviors for young adolescents. In addition, the survey data showed that some approaches were often used together (e.g., active and restrictive) and qualitative data similarly found that most parents did not have one monitoring strategy. Instead, strategies depended on the child and circumstances – and often involved all types of monitoring (e.g., giving free rein but also surveilling use or active communication with strict rules). Future research should explore how patterns of use, rather than specific strategies, relate to child outcomes.

### Limitations and Future Directions

This study is not without limitations. These data are cross-sectional, therefore prohibiting any causal interpretations of the results. Our survey sample was predominantly White, and findings may not generalize to parents of other racial, ethnic, and cultural groups. To increase understanding about parents from other cultural backgrounds, we used purposive sampling methods to increase the racial diversity of our sample as well as more balanced representation of families with sons versus daughters. The parents recruited for either survey or interview may have skewed more toward restrictive approaches, potentially due to social desirability or the need to report “good parenting” behaviors. Furthermore, parent reporting of perceived PIU may not represent what children might report themselves. Multi-informant responses in future studies can strengthen the interpretation of the results and the adolescent perspective. We also acknowledge the measurement of parental monitoring as single-indicator items, inconsistency of problematic internet versus device across parent and child use, and less reliability of the family closeness measure limits our ability to interpret the results with robust confidence. The measurement of the variable constructs was adapted from existing items, however not validated in the current form of the study thus creating opportunities for lower internal reliability.

This study was conducted prior to the COVID-19 pandemic, which has shifted the narrative of social technology use for adolescents. Future directions of this research should include considerations of longitudinal study design to further investigate the developmental nuances of using social technology during early adolescence through high school. The measurement of parental monitoring behaviors can also be expanded, especially in consideration of the findings from the qualitative portion of this study which included additional monitoring behaviors that are often not typically captured in the traditional measurement of media monitoring (e.g., surveillance, deference). A re-evaluation of existing media monitoring questionnaires can adapt to the rapidly evolving and dynamic nature of monitoring behaviors among parents as the ubiquity of device use expands to parents alongside early adolescents. Moreover, what is considered social technology is often contested, therefore, a measurement that captures all internet-based and social technology experiences can be beneficial for this field of research. Lastly, it is worth noting the increase in diversity of the youth population in the US context, therefore, a more sociodemographic representative sample of young adolescents and their parents will be critical in future studies to capture diverse characteristics of parental media monitoring and adolescent outcomes.

Practitioners who work with families dealing with social technology-related issues might consider the implications of our findings related to the types and range of media monitoring that relate to larger issues of family closeness. For instance, the finding that restrictive media monitoring is positively associated with parental perceptions of early adolescent’s problematic internet use indicates that parents’ perceptions of their child’s use may be related to their larger style of parental monitoring. Family closeness and parental device use are also important indicators of early adolescent online behaviors, such that practitioners might explore family communication strategies and modeling of social technology behaviors as part of a more holistic assessment of family technology contexts. Due to our findings that active and deference monitoring is associated with family contexts, but not early adolescent’s problematic online behaviors reveal the importance of ongoing dialog and a potential goal of allowing for more autonomous use built on trust over time. Our qualitative findings provided a window into the lived experiences of parents navigating an ill-defined parenting dilemma of monitoring social technology use. Practitioners can illuminate the common struggles that many families face to improve understanding of the many nuances of each approach, which do not happen in isolation. The qualitative findings also suggest that parental media monitoring strategies are constantly shifting depending on the child, context, and insecurities about effectiveness of their approaches, thus re-assessments over time to understand family technology contexts is critical.

## Conclusion

Young adolescents are increasingly engaging with social technologies and parents appear to be thinking deeply about how best to monitor that use. Using a mixed-methods approach, we shed light on how parents monitor their early adolescents’ digital media use and how parents’ own device use, and family technology context contribute to those monitoring behaviors. We found that both parents’ own problematic internet behaviors and feelings of family technoference were significantly related to their child’s problematic internet use. Further, parents describe similar strategies for different purposes. For instance, parents that perceived their child’s use to already be problematic were more likely to use restrictive strategies as an intervention, while parents who feared risky exposures online were also more likely to use restrictive strategies as prevention. Such findings suggest that parental monitoring of digital media use is likely a bidirectional process in which parents and children contribute to the strategies used. As early adolescence is a period of high device ownership and increasing transitions into social media platforms and connected spaces, understanding how their use is monitored and perceived by parents is important and provides initial steps into understanding trajectories of parental monitoring through adolescence. Parental monitoring is a well-established construct, and our data underscore its continued importance in our increasingly digitally connected world.

## Supplementary Information

JCFS_ParMon_Supplemental Materials_REVISION2
