# Supplementary material for: Parental Monitoring of Early Adolescent Social Technology Use in the US: A Mixed-Method Study
Source: J Child Fam Stud. 2023 Dec 18;33(3):759–76. doi: 10.1007/s10826-023-02734-6 (PMC12227363; doi:10.1007/s10826-023-02734-6)

Supplemental Materials

Figure S1. Parent-reported child and parent uses of social technology sites as an indicator of digital literacy
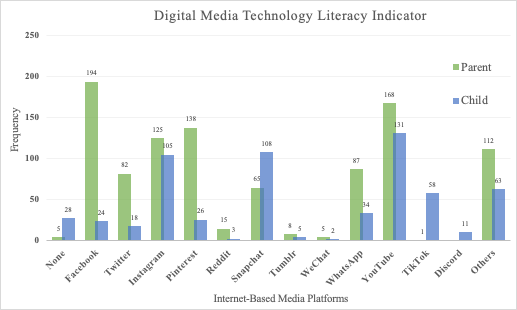


*Note*: Only 5 parents reported not using any of the internet-based media sites and 28 reported that their child does not use social media. TikTok and Discord use were only asked about for child’s internet-based media use. Other platforms included are *LinkedIn, House Party, ooVoo, Steam, VSCO,* and *Kik.*

Figure S2. Parent report of early adolescence social media use


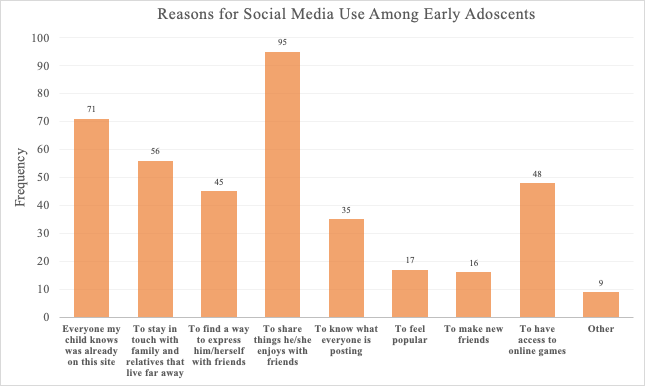


*Note*: “Other” includes communicating with friends without smartphones, making searches, accessing sports and health interests, watching videos, and promoting charity involvement.

Figure S3. Parental monitoring characteristics

(a) (b)
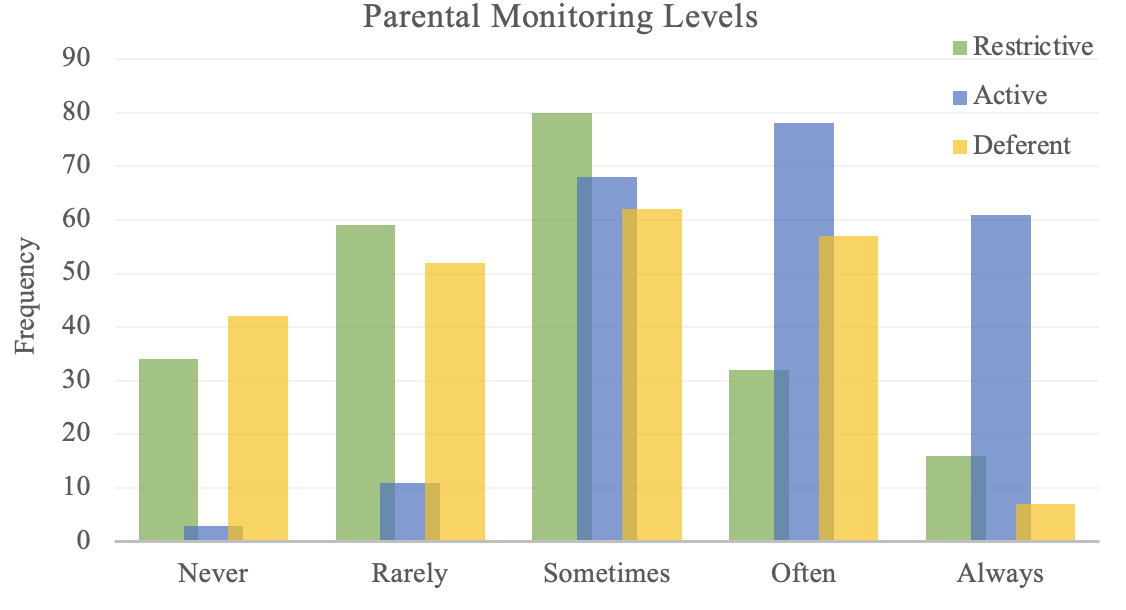


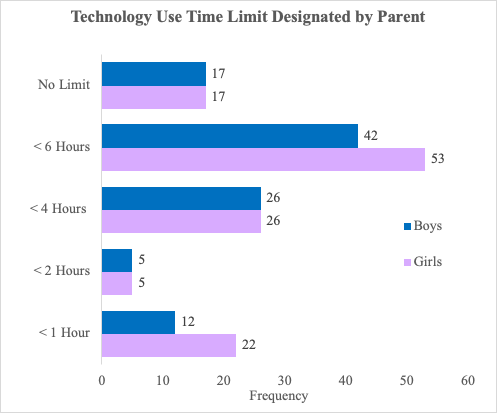


*Note*: Figure S3a frequencies collapsed by gender are as follows: < 1 hour = 15%; < 2 hours 4.4%; <4 hours = 22.9%; < 6 hour = 41.9%; and no limit = 15% of this sample.

Figure S4. Parental monitoring behaviors for young adolescents


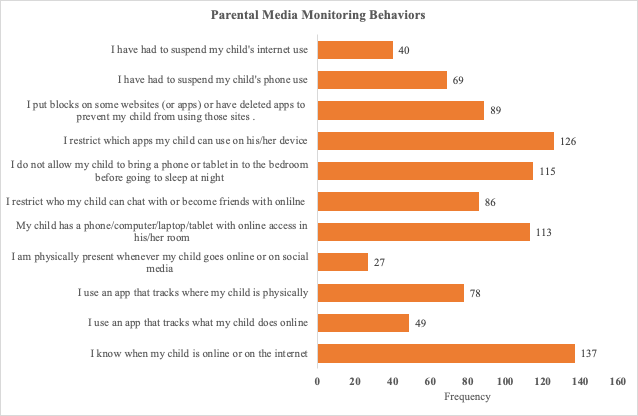

Supplement: Supplementary file 1 — JCFS_ParMon_Supplemental Materials_REVISION2 [file 10826_2023_2734_MOESM1_ESM.docx]
